# Patient enablement after a consultation with a general practitioner—Explaining variation between countries, practices and patients

**DOI:** 10.1111/hex.13091

**Published:** 2020-06-29

**Authors:** Elina Tolvanen, Peter P. Groenewegen, Tuomas H. Koskela, Torunn Bjerve Eide, Christine Cohidon, Elise Kosunen

**Affiliations:** ^1^ Faculty of Medicine and Health Technology c/o coordinator Leena Kiuru Tampere University Tampere Finland; ^2^ Pirkkala Municipal Health Centre Pirkkala Finland; ^3^ Science Centre Pirkanmaa Hospital District Tampere Finland; ^4^ Nivel—Netherlands Institute for Health Services Research Utrecht The Netherlands; ^5^ Department of Sociology Utrecht University Utrecht The Netherlands; ^6^ Department of Human Geography Utrecht University Utrecht The Netherlands; ^7^ Department of General Practice Institute of Health and Society University of Oslo Oslo Norway; ^8^ Department of Family Medicine Center for Primary Care and Public Health (Unisanté) University of Lausanne Lausanne Switzerland; ^9^ Centre for General Practice Pirkanmaa Hospital District Tampere Finland

**Keywords:** cultural dimensions, general practice, multi‐level modelling, patient enablement, primary health care

## Abstract

**Background:**

Patient enablement is a concept developed to measure quality in primary health care. The comparative analysis of patient enablement in an international context is lacking.

**Objective:**

To explain variation in patient enablement between patients, general practitioners (GPs) and countries. To find independent variables associated with enablement.

**Design:**

We constructed multi‐level logistic regression models encompassing variables from patient, GP and country levels. The proportions of explained variances at each level and odds ratios for independent variables were calculated.

**Setting and Participants:**

A total of 7210 GPs and 58 930 patients in 31 countries were recruited through the Quality and Costs of Primary Care in Europe (QUALICOPC) study framework. In addition, data from the Primary Health Care Activity Monitor for Europe (PHAMEU) study and Hofstede's national cultural dimensions were combined with QUALICOPC data.

**Results:**

In the final model, 50.6% of the country variance and 18.4% of the practice variance could be explained. Cultural dimensions explained a major part of the variation between countries. Several patient‐level and only a few practice‐level variables showed statistically significant associations with patient enablement. Structural elements of the relevant health‐care system showed no associations. From the 20 study hypotheses, eight were supported and four were partly supported.

**Discussion and Conclusions:**

There are large differences in patient enablement between GPs and countries. Patient characteristics and patients’ perceptions of consultation seem to have the strongest associations with patient enablement. When comparing patient‐reported measures as an indicator of health‐care system performance, researchers should be aware of the influence of cultural elements.

## 1 INTRODUCTION

Patients’ evaluation of care is a key element of the quality of health care. To study this, many patient‐reported outcome measures (PROMs) have been created.^1^ Most PROMs are disease‐specific and concern planned care.^2^ In primary care, the range of problems that patients present during consultations is unrestricted, a specific diagnosis is often not reached,^3^, ^4^ and a large part of care is unplanned. Therefore, a generic approach to PROMs is required. One such approach is patient enablement.

Patient enablement is a concept that was developed to measure quality of care, especially in primary care. It is defined as the patient's ability to understand and cope with illness and life after a consultation with a doctor.^5^ It could be measured using the Patient Enablement Instrument (PEI), a six‐item questionnaire addressed to a patient after a consultation.^5^ It is suggested that the PEI is a good PROM^5^, ^6^, ^7^ and it has been applied in several countries.^7^, ^8^, ^9^, ^10^, ^11^, ^12^, ^13^, ^14^, ^15^ Also, a single‐item measure has been shown to adequately identify patients with low enablement with high negative predictive value.^16^

**FIGURE 1:**
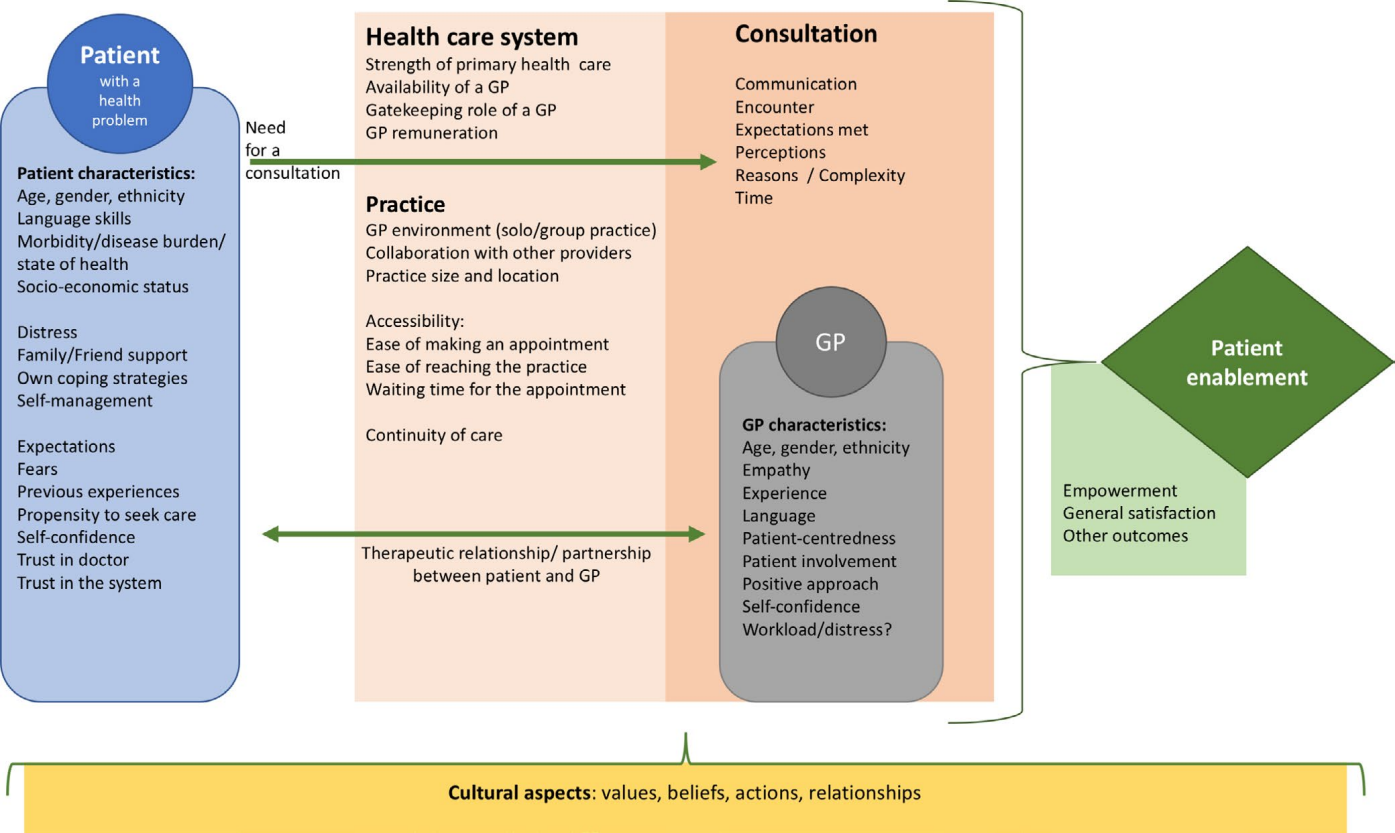
Patient enablement process

In previous studies, several factors are found to be associated with patient enablement. These could be divided into patient, consultation and system factors.^17^ Patient factors include patient characteristics, expectations and skills. Consultation factors include actions and perceptions of the consultation and general practitioner (GP) characteristics. System factors include organizational characteristics, such as characteristics of GP/practices or the structure of the health‐care system. A conceptual model of the process leading to patient enablement is presented in Figure 1.

When comparing separate studies, patient enablement seems to differ across countries. However, only one study directly compares patient enablement between countries^15^ and only a few report on comparisons of patient enablement between practices or doctors.^18^, ^19^, ^20^, ^21^, ^22^ Furthermore, to our knowledge there are no publications that consider the possible effect of cultural aspects on enablement. In other words, a comparative analysis to explain the differences in patient enablement between health‐care systems and countries is lacking.

The aim of this study is to explain variations in patient enablement between patients, GPs and countries. Based on the current literature, we have formulated hypotheses concerning the process of patient enablement. We test these hypotheses with a large international data set from 31 countries, using multi‐level modelling. We use a single‐item measure as an indicator of patient enablement. To our knowledge, this is the first study of patient enablement that takes the differences between health‐care system features or cultural aspects into consideration.

## 2 HYPOTHESES

In the following sections, we present the current knowledge on factors associated with enablement and hypothesize the mechanisms behind these associations. Consequently, we formulate our study hypotheses.

### 2.1 Patient‐level hypotheses

#### 2.1.1 Patient characteristics

At the patient level, it could be suggested that ‘who the patient is and how they act’ is essential to how patients evaluate the consultation. Previous results are contradictory regarding age^7^, ^8^, ^9^, ^10^, ^19^, ^20^ and gender.^7^, ^19^, ^20^ With the exception of one study,^8^ neither education nor income has shown any association with enablement.^7^, ^17^

Hypothesis 1 Patient age, gender or socio‐economic status is not associated with patient enablement.

Consultation in the patient's native language seems to promote enablement.^23^ On the other hand, immigrants have reported higher enablement scores than natives in the UK.^20^, ^24^, ^25^ Patients’ culturally conditioned attitudes towards authorities (eg doctors) might influence the way patients evaluate the consultation.

Hypothesis 2a Patients’ non‐immigrant background is associated with lower enablement.

Hypothesis 2b Patients’ weak language skills are associated with lower enablement.

Considering patient health, lower self‐perceived health,^8^, ^17^, ^19^ the presence of a chronic illness^7^, ^22^ or multimorbidity^17^ has been associated with lower enablement.

Hypothesis 3 The presence of chronic illness or lower self‐perceived health is associated with lower enablement.

#### 2.1.2 Patient‐perceived consultation factors

It is likely that enablement increases when patients can understand their doctor and feel confident that their collaboration functions well. Patients’ positive perceptions regarding doctor‐patient communication^7^, ^25^, ^26^, ^27^ as well as involvement in decision making^15^ have been associated with higher levels of enablement. Furthermore, patient satisfaction has shown a rather strong positive association with enablement.^20^, ^22^, ^28^, ^29^

Hypothesis 4 Negative perceptions of communication or patient involvement are associated with lower enablement.

Hypothesis 5 Lower patient satisfaction is associated with lower enablement.

In general, enablement may be higher when there is a clear problem to solve in the consultation. Having an appointment due to long‐standing conditions^17^ or complex reasons^5^, ^30^ is found to be associated with lower enablement.

Hypothesis 6 A consultation for a long‐standing condition is associated with lower enablement.

Although there are no studies about previous experiences of health care and enablement, we expect that previous negative experiences are associated with lower enablement.

Hypothesis 7 Previous negative experiences of health care are associated with lower enablement.

Patients’ trust in the doctor seems to promote enablement,^31^ and we also expect it to apply in this study. In addition, particularly in non‐gatekeeping primary care systems, the fact that patients visit a GP instead of another specialist might reflect their confidence in a GP. Thus, we expect that a patient's propensity to seek care from a GP might promote enablement.

Hypothesis 8 Lower trust in the doctor is associated with lower enablement.

Hypothesis 9 Lower propensity to seek care from a GP is associated with lower enablement.

#### 2.1.3 Patient‐perceived system factors

Better continuity of care, especially when patients know the doctor, tends to support higher enablement.^7^, ^8^, ^11^, ^20^, ^24^, ^26^, ^32^, ^33^ It seems reasonable to hypothesize that if the patient and the doctor know each other, and particularly if the relationship is good, enablement after an appointment is easier to achieve. In addition, poorer access to care, as indicated by longer waiting times, seems to be associated with lower enablement.^34^

Hypothesis 10 Weaker continuity of care is associated with lower enablement.

Hypothesis 11 Weaker access to care is associated with lower enablement.

### 2.2 GP‐/practice‐level hypotheses

#### 2.2.1 GP and practice characteristics

It seems reasonable to hypothesize that GP characteristics are important for enablement. However, current knowledge about such associations is scarce. A GP’s age and gender have shown to have either partial^8^ or no effect^7^ on patient enablement in previous studies. In addition, organizational structure might relate to practice outcomes. GPs working in single‐handed practices^20^ or those that have a medium‐sized patient list^21^ have been associated with higher patient enablement. Results related to patient enablement in relation to GP workload are contradictory.^8^, ^22^ Furthermore, we suggest that salaried GPs have less incentive to enable patients. Practice location may have an impact on continuity of care ^35^, ^36^ and thus be associated with enablement.

Hypothesis 12 GP’s age and gender have no association with patient enablement.

Hypothesis 13 GP’s practice accommodation (duo or group practice), remuneration (salaried GPs) or practice location (rural) is associated with lower enablement.

Hypothesis 14 GP’s perception of high workload or work‐related stress is associated with lower patient enablement.

#### 2.2.2 Practice‐related consultation characteristics

Among practice‐related consultation characteristics, the length of the consultation is probably the most studied factor, revealing that longer consultations are associated with higher enablement.^5^, ^20^, ^25^, ^30^, ^33^, ^34^, ^37^ Associations of other practice‐related consultation characteristics with patient enablement have not been studied. We expect that GPs who have opportunities to do more varied work, for example by performing technical procedures, collaborating with other providers and thus taking care of their patients more extensively, may enable patients better.

Hypothesis 15 Shorter consultation times are associated with lower enablement.

Hypothesis 16 A lack of opportunities for GPs to collaborate with other providers or perform technical procedures is associated with lower patient enablement.

### 2.3 Country‐level hypotheses

#### 2.3.1 Health‐care system characteristics

The structural strength of primary health care could be assessed from three dimensions: governance, economic conditions and workforce development.^38^, ^39^ In this study, we expect that a weaker primary care structure will reduce expectations towards GPs and thus lead to lower enablement. Furthermore, in gatekeeping countries, the GP is usually the first contact in health care. This could promote continuity of care and thus enablement.

Hypothesis 17 A weaker primary health‐care structure is associated with lower enablement.

Hypothesis 18 Enablement is lower in non‐gatekeeping countries.

#### 2.3.2 Cultural dimensions

Culture could be defined as ‘the customary beliefs, social forms and material traits of a racial, religious or social group’; or ‘the integrated pattern of human behaviour that includes thought, speech, action and artefacts’.^40^ Indeed, culture may have an impact on our actions and feelings, and shape what we value in health care.^41^, ^42^, ^43^, ^44^ For example, in a study conducted in eight countries, the statement ‘during the consultations a GP should have enough time to listen, talk and explain to me’ was ranked very/most important by 85%‐93% of the respondents.^42^ In contrast, the statement ‘it should be possible to see the same GP at each visit’ was ranked rather important in Norway (rank 6 of 38) and significantly less important in the UK (rank 28 of 38).^42^

In an analysis of the QUALICOPC data for Switzerland, enablement was linked with the linguistic area.^22^ Otherwise, there are no publications that link patient enablement with cultural differences. Cultural differences in doctor‐patient relationships might have an effect on enablement. In some countries, doctors are seen more as authorities, whereas in others doctors are seen more as equals. Furthermore, in cultures with a stronger emphasis on individual than societal values, patients might be more difficult to satisfy, and this might lead to lower enablement.

Hypothesis 19 Patient enablement is lower in countries with less emphasis on patient enablement.

Hypothesis 20 Cultural dimensions are associated with enablement: a greater power distance and more emphasis on individual values are associated with lower enablement.

## 3 METHODS

### 3.1 Population

In this study, we use the data collected in the Quality and Costs of Primary Care in Europe (QUALICOPC) study. The details of the QUALICOPC study design and data collection are described elsewhere.^45^, ^46^, ^47^ The purpose of the QUALICOPC study is ‘to evaluate the system, the practice and the patient’ by studying different primary care systems in 31 European countries, along with Australia, Canada and New Zealand. The goal was to reach 75 GPs in Cyprus, Iceland, Luxembourg and Malta, and 220 in all other countries. Only one GP per practice could participate in the study. For each GP, the goal was to recruit nine patients to fill in the Patient Experience Questionnaire and one patient to fill out the Patient Values Questionnaire.^46^ Patients were recruited in the GPs’ waiting room.

#### 3.1.1 Measurements and data

In this study, patient enablement was measured using a single question ‘After this visit, I feel I am able to cope better with my symptom/illness than before the appointment’, with possible answers being yes/no/don't know. The don't knows were combined with the no responses. When compared with the Patient Enablement Instrument, which is considered the gold standard for measuring patient enablement, this question seems to adequately identify patients with low enablement.^16^

Operationalization of the concepts used as independent variables is presented in File S1. Some of the constructs were operationalized through scale variables. These scales were calculated using the ecometric approach, in which multi‐level analysis is used to construct a contextual variable at a higher‐level unit based on individual variables. The scale construction process has been used in previous studies using QUALICOPC data and is described in detail elsewhere.^48^ To improve interpretability of the models, the scale scores were transformed into z‐scores (score minus the average divided by the standard deviation); hence, a score of 0 represents the mean score and a score of 1 represents one standard deviation increase.

We also used data from the Primary Health Care Activity Monitor for Europe (PHAMEU) study^49^ to include country‐level variables regarding primary care dimensions. The PHAMEU dimensions included in this study are governance, economic conditions, workforce development and total structure.^38^

In addition, we used Hofstede's dimension model of national cultures, based on a data set originally collected from employees of a multinational corporation,^50^ applied in 111 countries.^51^ The model consists of six dimensions that reflect societal tendencies of (1) people to feel independent instead of interdependent (individualism vs. collectivism); (2) attitudes towards unequal power distribution (power distance); (3) social endorsement for use of force (masculinity vs. femininity); (4) tolerance of uncertainty and ambiguity (uncertainty avoidance); (5) attitudes towards change (long‐term vs. short‐term orientation); and (6) attitudes towards good things in life (indulgence vs. restraint).^50^, ^51^ More detailed explanations of these dimensions are presented in File S2. In Hofstede's model, each nation has a unique combination of these six dimensions, reflecting stable cultural values of the society.

The original QUALICOPC data set includes a total of 34 countries, whereas Hofstede's data do not include Cyprus, Iceland and FYR Macedonia. In order to maintain comparability between the different models, these three countries were left out of the analyses.

### 3.2 Statistical analyses

Due to the collection method, the structure of the QUALICOPC data is hierarchically clustered, meaning that patients are nested within their GPs and the GPs are nested within countries, forming three levels: patient, GP and country levels. With this kind of data, multi‐level modelling should be used.^52^ Multi‐level modelling allows the analysis of individual‐level outcomes in relation to variables at the same or higher levels and to split up the total variation in an outcome variable into parts that are attributable to the different levels.^53^

**FIGURE 2:**
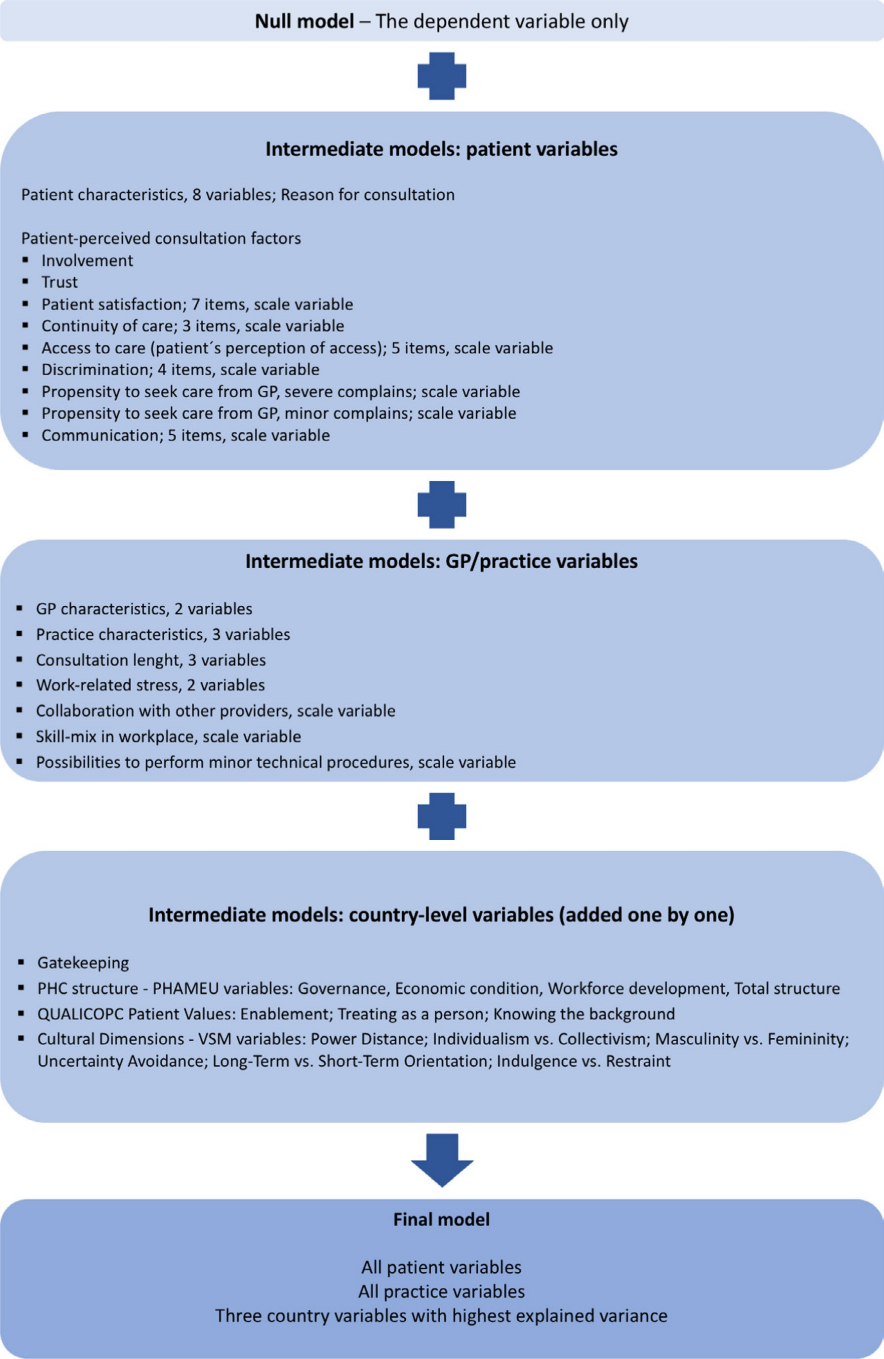
The modelling strategy

Multi‐level, multivariable logistic regression models were constructed in order to explain variations in patient enablement between patients, practices/GPs and countries, and to find significant factors associated with lower enablement. The modelling strategy is presented in Figure 2. First, ‘a null model’ (Model 0) was performed to explore variances between countries and practices. To calculate the share of variance at practice and country levels, individual‐level variance was approximated by pi^2^/3. Second, patient‐level variables (patient characteristics and patient perceptions of the consultation) were included (Model 1). Next, practice‐level variables (GP and practice characteristics) were added to Model 1 (Model 2). Finally, country‐level variables (health‐care system characteristics, primary care dimensions and cultural dimensions) were added one by one. Three country‐level variables that could best explain the variation were then retained in the final model (Model 3). The explanatory power of the models was evaluated by calculating the explained variance of each model compared to the variance in the null model.

Also, median odds ratios (MORs) were calculated for each model. The MOR is the median odds ratio between two randomly chosen individuals with the same covariates but from different clusters.^54^ When using this approach, differences in probability/risk are entirely quantified by the cluster‐specific effects.^54^, ^55^ The MOR is comparable with individual‐level ORs and thus helps to quantify the extent of clustering.^55^

As the number of higher‐level variables should not exceed 10% of the number of higher‐level units,^53^ only three country‐level variables could be included simultaneously in the final model. Missing values were excluded from the analyses. For two variables (trust in doctors in Australia and Poland and mean consultation time in Australia), there were no observations. Thus, value imputation (replacing the missing value by an average value of the subset of other countries) was used in order to minimize the loss of data.

## 4 RESULTS

Data collected from a total of 7210 GPs from 31 countries were used in this analysis. From the practices of these GPs, 61 458 patients were recruited to participate. The distributions of patient and GP characteristics are presented in Tables 1 and 2. Among the participants, 58 930 patients answered the dependent variable ‘After this visit, I feel I am able to cope better with my symptom/illness than before the appointment’. Some 13 367 (21.7%) answered ‘no’ or ‘don't know’, interpreted as lower enablement. Table 3 presents the distribution of the dependent variable in each country. The distributions varied largely between countries: for example, the proportion of lower enablement varied from 9.2% in New Zealand to 39.6% in Sweden.

**TABLE 1:** Distribution of patient characteristics, n = 61 458

	n	%
Age		
17‐39	18 024	29.3
40‐64	27 330	44.5
65 or over	15 061	24.5
Missing	1043	1.7
Gender		
Male	23 735	38.6
Female	37 257	60.6
Missing	466	0.8
Household income		
Below average	18 428	30.0
Around average	34 487	56.1
Above average	7573	12.3
Missing	970	1.6
Education		
No qualifications obtained/pre‐primary education or primary	16 529	26.9
Upper secondary level of education	23 147	37.7
Post‐secondary, non‐tertiary education	20 655	33.6
Missing	1127	1.8
Ethnicity		
Native	53 369	8.8
Second‐generation immigrant	2624	4.3
First‐generation immigrant	4837	7.9
Missing	628	1
Language skills		
Fluently/native speaker level	49 086	79.9
Sufficiently	11 618	18.9
Missing	754	1.2
Chronic disease		
No	30 582	49.8
Yes	30 505	49.6
Missing	371	0.6
Self‐perceived health		
Very good	37 301	60.7
Poor	23 875	38.9
Missing	277	0.5
Consultation reason		
Illness	22 958	37.4
Medical check‐up	15 001	24.4
Prescription, certificate or referral	12 123	19.7
Other	11 054	18.0
Missing	313	0.5

**TABLE 2:** Distribution of GP characteristics, n = 7120

	n	%
Age		
21‐39	1095	15.4
40‐64	5578	78.3
65 or over	370	5.2
Missing	77	1.1
Gender		
Male	3395	47.7
Female	3697	51.9
Missing	28	0.4
Practice location		
Large (inner city)	2137	30.4
Suburbs or small town	2477	35.2
Urban‐rural or rural	2424	34.4
Missing	82	1.2
GP accommodation		
Solo practice	2856	40.1
Duo or group practice	4194	58.9
Missing	70	1.0
GP remuneration		
Salaried	2324	32.6
Self‐employed	4621	64.9
Mixed	72	1.0
Missing	103	1.5
GP‐perceived work‐related stress		
Agree	4073	57.2
Disagree	2953	41.5
Missing	94	1.3
GP‐perceived effort‐reward balance		
Agree	3354	47.1
Disagree	3676	51.6
Missing	90	1.3
Mean consultation time (minutes, GP estimate)		
Mean	14.5	
SD	7.1	
Range	0‐120	
Missing	240	
Mean number of face‐to‐face consultations per day (GP estimate)		
Mean	30.7	
SD	16.0	
Range	0‐88	
Missing	49	

**TABLE 3:** Distribution of the dependent variable ‘After this visit, I feel I am able to cope better with my symptom/illness than before the appointment’, by country, n = 61 458

	No + don't know		Yes		Missing		Total
	N	%	N	%	N	%	N
Austria	276	17.3	1216	76.2	104	6.5	1596
Belgium	856	23.3	2611	71.1	207	5.6	3674
Bulgaria	611	30.9	1331	67.4	33	1.7	1975
Czech Republic	454	22.9	1500	75.7	28	1.4	1982
Denmark	333	17.7	1407	74.8	140	7.4	1880
Estonia	325	28.9	754	67.0	47	4.2	1126
Finland	269	20.0	900	66.9	**177**	**13.2**	1346
Germany	391	18.5	1683	79.5	44	2.1	2118
Greece	461	23.6	1474	75.4	21	1.1	1956
Hungary	636	32.9	1213	62.7	87	4.5	1936
Ireland	184	11.0	1299	77.4	196	11.7	1679
Italy	363	18.6	1474	75.5	116	5.9	1953
Latvia	577	29.8	1297	67.0	63	3.3	1937
Lithuania	572	28.4	1428	70.9	**13**	**0.6**	2013
Luxembourg	133	18.7	531	74.8	46	6.5	710
Malta	103	16.5	511	81.6	12	1.9	626
Netherlands	649	32.6	1170	58.8	172	8.6	1991
Norway	523	34.1	889	58.0	121	7.9	1533
Poland	505	25.6	1457	73.8	**12**	**0.6**	1974
Portugal	240	12.8	**1598**	**85.0**	43	2.3	1881
Romania	413	20.9	1547	78.3	16	0.8	1976
Slovakia	672	35.1	1159	60.5	85	4.4	1916
Slovenia	521	24.0	1571	72.4	79	3.6	2171
Spain	778	20.9	2882	77.3	69	1.9	3729
Sweden	**310**	**39.6**	**398**	**50.8**	75	9.6	783
Switzerland	368	20.5	1389	77.5	35	2.0	1792
Turkey	499	19.1	2100	80.3	**15**	**0.6**	2614
UK	237	18.1	949	72.4	124	9.5	1310
Australia	125	10.3	1022	84.5	62	5.1	1209
Canada	874	12.5	5828	83.6	270	3.9	6972
New Zealand	**109**	**9.2**	975	81.9	106	8.9	1190
Total	13 367	21.7	45 563	74.0	2618	4.3	61 548

Note:

Lowest and highest proportion of each answer are bolded.

### 4.1 Multi‐level modelling—explaining variation

The model variances, proportions of explained variances and the median odds ratios (MORs) for each level are presented in Table 4. In the null model, 16% of the variance is at practice level and 6% at country level. For ease of interpretation of the amount of variation at the different levels, we also calculated the median odds ratios (MORs) for practice and country levels. These were 2.01 and 1.41, respectively, and can be compared to the odds ratios of the independent variables. Thus, the effect of the clusters (the differences between practices or countries) in enablement is greater than the effect of most of the independent variables. After adding all patient‐level variables, the model explained only 0.96% of country variation and 20.3% of practice variation. In addition, almost all patient variables in the model had a statistically significant association with the dependent variable. Since having all the variables in the model explained a higher proportion of the variances, all the variables were kept in the model.

**TABLE 4:** Model variances, explained variances and median odds ratios (MORs) for each level

Model variances	Null model	Model 1	Model 2	Model 3 Final model
Country variance	0.2598	0.2573	0.2230	0.1284
Practice variance	0.661	0.5264	0.5398	0.5398
Country variance explained, %		0.96	14.2	50.6
Practice variance explained, %		20.3	18.4	18.4
MOR (median odds ratio) for country level	1.63	1.62	1.56	1.41
MOR for practice level	2.17	2.00	2.01	2.01

Adding GP/practice variables to the model decreased the proportion of explained practice variance, reflecting that the true practice variance was masked in the simpler model. In addition, it increased the explained country variance to 14.2%. Thus, all GP‐level variables were kept in the model.

Finally, country variables were added one by one, and those that explained the highest proportion of country variance were included in the final model. The three country variables best explaining the country‐level variation were all cultural dimensions: individualism vs. collectivism, uncertainty avoidance and long‐term orientation. None of the structural elements of primary care system were good explainers. Comparisons of country‐level variables are presented in Table 5. With the final model, 50.6% of the country variance and 18.4% of the practice variance could be explained.

**TABLE 5:** Summary of the study hypotheses and the results of the logistic regression analysis in the final model: the odds ratio (OR) to respond negatively to the dependent question ‘After this visit, I feel I can cope better with my symptom/illness than before the appointment’

Patient‐level hypothesis	OR	p	95%CI	Conclusion for hypothesis
**H1. Patient's age, gender or socio‐economic status is not associated with patient enablement**				**Rejected**
Patient's age: Under 40 y (ref)				
40‐64 y	**0.84**	**<0.001**	**0.79‐0.89**	
Over 65 y	**0.81**	**<0.001**	**0.73‐0.90**	
Patient's gender: Male (ref)				
Female	**0.87**	**<0.001**	**0.83‐0.92**	
Education: No/primary level (ref)				
Upper secondary level	1.04	0.25	0.97‐1.11	
Post‐secondary level	**1.09**	**0.03**	**1.01‐1.18**	
Household income: Below average (ref)				
Around average	**0.91**	**0.003**	**0.86‐0.97**	
Above average	0.93	0.15	0.85‐1.02	
Occupation: Working, including civil service and self‐employment (ref)				
Retired	0.93	0.13	0.85‐1.02	
Student, unemployed, unable to work, mainly homemaker	**1.07**	**0.04**	**1.00‐1.14**	
**H2a. Patient's non‐immigrant background is associated with lower enablement.**				**Rejected**
Ethnicity: Native (ref)				
Second‐generation immigrant	1.07	0.28	0.95‐1.21	
First‐generation immigrant	0.90	0.07	0.81‐1.01	
**H2b. Patient's weak language skills are associated with lower enablement.**				**Rejected**
Language skills: Fluently/native speaker level (ref)				
Sufficiently/moderately/poorly/not at all	1.01	0.89	0.93‐1.09	
**H3a. Lower self‐perceived health is associated with lower enablement.**				**Supported**
Self‐perceived health: Very good/good (ref)				
Fair/poor	**1.29**	**<0.001**	**1.22‐1.37**	
**H3b. The presence of chronic illness is associated with lower enablement.**				**Rejected**
Chronic disease: No (ref)				
Yes	0.98	0.61	0.93‐1.05	
**H4a. Negative perception of patient involvement is associated with lower enablement.**	**Supported**			
Patient involvement: No (ref)				
Yes	**0.58**	**<0.001**	**0.54‐0.62**	
**H4b. Negative perception of communication is associated with lower enablement**				**Rejected**
Positive perception of communication (scale with 5 variables)	1.03	0.07	0.99‐1.07	
**H5. Lower patient satisfaction is associated with lower enablement.**				**Supported**
Positive patient satisfaction (scale with 7 variables)^a^	**0.54**	**<0.001**	**0.52‐0.56**	
**H6. A consultation for a long‐standing condition is associated with lower enablement.**				**Rejected**
Consultation reason: Illness (ref)				
Medical check‐up	1.06	0.08	0.99‐1.13	
Prescription, referral or certificate	**1.40**	**<0.001**	**1.31‐1.51**	
Other	**1.20**	**<0.001**	**1.11‐1.29**	
**H7. Previous negative experience of health care is associated with lower enablement.**				**Supported**
No previous experience of discrimination (scale with 4 variables)^a^	**0.96**	**0.002**	**0.93‐0.98**	
**H8. Lower trust in the doctor is associated with lower enablement.**				**Supported**
Trust in doctors in general: Agree (ref)				
Disagree	**1.58**	**<0.001**	**1.41‐1.77**	
**H9. Lower propensity to seek care from a GP is associated with lower enablement**				**Supported**
Propensity to seek care (severe complains, scale)^a^	**0.86**	**<0.001**	**0.83‐0.88**	
Propensity to seek care (minor complains, scale)^a^	**0.89**	**<0.001**	**0.86‐0.91**	
**H10. Weaker continuity of care is associated with lower enablement.**				**Supported**
Continuity of care (scale with 3 variables)^a^	**0.70**	**<0.001**	**0.67‐0.73**	
**H11. Weaker access to care is associated with lower enablement**				**Supported**
Positive perceptions of access to care (scale variable with 5 variables)^a^	**0.84**	**<0.001**	**0.81‐0.87**	
*GP‐level hypotheses*				
**H12. GP’s age and gender are not associated with enablement.**				**Supported**
GP’s age: 21‐39 (ref)				
40‐64	1.05	0.29	0.96‐1.15	
65 and over	1.09	0.32	0.92‐1.28	
GP gender: Male (ref)				
Female	0.98	0.53	0.92‐1.05	
**H13a. GP’s practice location is associated with enablement.**				**Supported**
GP practice location: Large inner city (ref)				
Suburbs or small town	1.08	0.07	0.99‐1.17	
Urban‐rural or rural	**1.12**	**0.01**	**1.03‐1.22**	
**H13b. GPs’ practice accommodation (duo or group practice) and remuneration (salaried GPs) are associated with lower enablement**				**Rejected**
GP accommodation: Solo practice (ref)				
Duo or group practice	0.98	0.58	0.91‐1.06	
GP remuneration: Salaried (ref)				
Self‐employed	1.11	0.08	0.99‐1.24	
Mixed	0.92	0.63	0.64‐1.30	
**H14. GP’s perception of high workload or work‐related stress is associated with lower enablement.**				**Rejected**
GP‐perceived work‐related stress: Agree				
Disagree	1.03	0.43	0.96‐1.10	
GP‐perceived effort‐reward imbalance: Agree				
Disagree	1.00	1.00	0.93‐1.07	
**H15. Shorter consultation time is associated with lower enablement**				**Rejected**
Mean consultation time (GP estimation): 0‐4 min (ref)				
5‐9 min	0.82	0.21	0.60‐1.11	
10‐14 min	0.82	0.19	0.60‐1.11	
15‐29 min	0.76	0.09	0.56‐1.04	
Over 30 min	0.71	0.05	0.50‐1.01	
Mean number of face‐to‐face consultations per day (GP estimation): 0‐14 (ref)				
15‐29	0.91	0.19	0.80‐1.04	
30‐44	0.91	0.18	0.78‐1.05	
45 or more	**0.82**	**0.02**	**0.70‐0.97**	
**H16. A lack of opportunities for GPs to collaborate with other providers or perform technical procedures is associated with lower enablement.**				**Rejected**
Collaboration with other providers (scale)^a^	1.02	0.38	0.98‐1.06	
Occupational skill mix in workplace (scale)^a^	0.96	0.25	0.88‐1.03	
Possibility to perform technical procedures (scale)^a^	1.00	0.98	0.95‐10.6	
*Country‐level hypotheses* *Note: Country‐level variables were included in the model one by one*				
**H17. Weaker primary health‐care structure is associated with lower enablement.**				**Rejected**
PHC structure—PHAMEU variables				
Governance	1.02	0.78	0.87‐1.19	
Economic condition	1.09	0.28	0.93‐1.28	
Workforce development	0.96	0.66	0.80‐1.15	
Total structure	1.02	0.81	0.86‐1.20	
**H18. Enablement is lower in non‐gatekeeping countries.**				**Rejected**
Gatekeeping (referred to non‐gatekeeping countries)	1.46	0.15	0.92‐1.80	
**H19. Patient values are associated with enablement: enablement is lower in countries with less emphasis on patient enablement.**				**Rejected**
‘It is important that I can cope better after the appointment’	0.87	0.13	0.73‐1.04	
‘It is important that the doctor treats me as a person and not just a medical problem’	1.04	0.68	0.86‐1.26	
‘It is important that this doctor knows important information about my medical background’	0.93	0.39	0.77‐1.10	
**H20. Cultural dimensions are associated with enablement: larger power distance and more emphasis on individual values are associated with lower enablement.**				**General hypothesis supported**
Power distance	0.88	0.14	0.75‐1.04	**Rejected**
Individualism vs. collectivism	**1.21**	**0.03**	**1.02‐1.43**	**Rejected**
Masculinity vs. femininity	0.87	0.08	0.72‐1.02	
Uncertainty avoidance	**0.84**	**0.03**	**0.72‐0.99**	
Long‐term vs. short‐term orientation	**1.26**	**0.003**	**1.08‐1.46**	
Indulgence vs. restraint	0.98	0.81	0.82‐1.16	
**The ORs of the three best variance explaining variables in the final model, all patient and GP variables included**				
Individualism vs. collectivism (towards individualism)	1.11	0.26	0.93‐1.32	
Uncertainty avoidance (towards uncertainty avoiding)	0.88	0.15	0.74‐1.04	
Long‐term orientation (towards short‐term orientation)	**1.27**	**<0.001**	**1.11‐1.46**	

Note

Statistically significant ORs are bolded.

^a^ Scale variables are presented as *z*‐scores.

### 4.2 Logistic regression—evaluating associations

Several independent variables had statistically significant associations with the dependent variable, i.e. lower enablement. Table 5 presents the results of the final multi‐level logistic regression model and the conclusions for the study hypotheses. Of the 20 study hypotheses, eight were rejected and eight supported, and four of the hypotheses were partly supported and partly rejected. Also, File S3 includes all the logistic regression results of Models 1–3, the level variances and the median odds ratios (MORs) in each model.

When regarding patient‐level variables, patients with a household income of around average, as well as older and female patients, had a smaller risk of lower enablement. Furthermore, positive perception of patient involvement, patient satisfaction, continuity of care, access to care, no discrimination and propensity to seek care from a GP were associated with a decreased risk of lower enablement. The strongest associations with decreased risk of lower enablement were found for positive patient satisfaction (OR 0.54, *P* < .001, 95%CI 0.52‐0.56) and positive perception of patient involvement (OR 0.58, *P* < .001, 95%CI 0.54‐0.62). In contrast, poorer self‐perceived health (OR 1.29, *P* < .001, 95%CI 1.22‐1.37) or higher educational level was associated with higher risk of lower enablement. Patients who were not working or retired (students, unemployed patients, patients unable to work due to illness and homemakers), or patients whose reason for consultation was due to prescription, certificate or referral on categorized as ‘other’, were more likely to report lower enablement. In addition, patients who reported having a lack of trust in doctors in general had increased risk of lower enablement (OR 1.58, *P* < .001, 95%CI 1.41‐1.77).

From the GP‐/practice‐level variables, a higher number of face‐to‐face consultations were associated with a decreased risk of lower enablement (OR 0.82, *P* = .02, 95%CI 0.70‐0.97), whereas a mixed urban‐rural or rural practice location was associated with an increased risk of lower enablement (OR 1.12, *P* = .01, 95%CI 1.03‐1.22). From three country‐level variables in the final model, only long‐term orientation had a statistically significant association with the dependent variable (OR 1.27, *P* < .001, 95%CI 1.11‐1.46). This indicates that patients in more long term–oriented cultures have a decreased risk of lower enablement.

## 5 DISCUSSION

In this study, we found that patient enablement, measured by a single question, varies largely between 31 countries. By using multivariable, multi‐level models, this variation between countries could be explained to a rather large extent. The logistic regression results of this study show that, for example, patient's older age, female gender and positive perceptions of patient satisfaction and patient involvement are associated with decreased risk of lower enablement. In contrast, for example, patient's worse self‐perceived health, reason for consultation and lower trust in doctors are associated with increased risk of lower enablement.

In general, patient characteristics and patients’ perception of the consultation do not explain the variation between countries. However, they do explain variance between practices to some extent. Furthermore, although adding GP‐level variables to the models improved it, the overall explained practice variance remained rather low—over 80% of variance remained unexplained. It is possible that the variables available in the QUALICOPC framework may not have included all the potentially important factors related to practices and GPs. In particular, the personal characteristics of a GP could have a strong influence on enablement; it is assumed that there are ‘high enablers’ and ‘low enablers’ among GPs.^20^

None of the PHAMEU structural elements of the health‐care system explained enablement variation between countries, contrary to our hypothesis. None of them was statistically associated with enablement. Thus, it seems that the mechanisms behind patient enablement are not system‐associated but more culture‐associated.

The cultural dimension, long‐term orientation, was the only country‐level variable that had a statistically significant association with patient enablement. According to the results of this study, people in more long term–oriented cultures have a decreased risk of lower enablement. This cultural dimension deals with change; in long term–oriented cultures, ‘the basic notion of the world is that it is in flux, and preparing for the future is needed’.^51^ In short term–oriented cultures, ‘the world is essentially as is was created, so the past provides a moral compass’.^51^ To our knowledge, there is no other evidence of a role of this dimension in the health‐care context. Perhaps people in more long term–oriented cultures adopt a more flexible attitude to changes in health as well.

The fact that the structure of the primary care system is not related to enablement, but a dimension of national culture is, has implications for the international comparison of PROMs. Before using PROMs as indicators for health system performance, the relationships with specific characteristics of health systems on the one hand and cultural characteristics on the other should be further explored. Previous research has shown that cultural values are related to different aspects of primary care.^56^

Patient characteristics show rather strong associations with patient enablement. In particular, a patient's age and gender have a clear association with patient enablement, even after adjusting for several other variables. This is against the a priori expectations which were based on contradictory results in the previous literature. However, in a large systematic review, older age is related to higher patient satisfaction,^57^ and the mechanism behind achieving enablement might be similar. It may be that young patients are more critical of care than the elderly, leading to lower enablement. In addition, elderly patients may have built a relationship with their GPs, after seeing them more often, and thus more easily experience enablement. Furthermore, women tend to have a more active attitude towards treatment and health,^58^ and this could also promote reported enablement following consultation.

The patients’ perception of a consultation seems to play a role in the enablement process. As expected, positive perceptions of the doctor‐patient relationship (eg involvement and continuity of care) decreased the risk of poorer enablement. Previous evaluations of doctors’ patient‐centeredness,^27^, ^33^ partnership with the patient ^26^ or patient satisfaction^20^, ^22^, ^28^, ^29^ have suggested positive associations with enablement. Furthermore, it is encouraging to find that the propensity to seek care from GPs significantly decreased the risk of poorer enablement—possibly a reflection of patients’ trust in primary health care. Against expectations, the patient's perception of communication was not associated with enablement in our study.

Two of our five GP‐level hypotheses were confirmed. As expected, GP’s age and gender were not associated with patient enablement. Instead, practice location played a role: more rural location was associated with a higher risk of lower enablement. This could be due to different patients and problems in rural compared to urban areas. Also, poorer continuity may have an effect: for instance, a Norwegian study showed that continuity was better in larger and usually more central municipalities.^36^ Better resources and access to care in more urban areas might be one reason for this result. In addition, the doctors (n = 1331) who meet more patients during a regular workday (over 45 compared to less than 15 patients) tend to enable their patients more than their colleagues with fewer daily patient contacts. This is contrary to the evidence ^5^, ^20^, ^25^, ^30^, ^33^, ^34^, ^37^ that a longer consultation time promotes enablement—the mechanism behind this result must be something other than just the minutes spent. Perhaps in systems where the GPs have as many as 45 consultations per day, patient has different expectations towards consultations. Also, the reasons for an encounter may be simpler in these systems.

## 6 STRENGTHS AND LIMITATIONS

A strength of this study is the large sample of GPs and their patients from many countries. Use of multi‐level modelling with this kind of data is necessary—the robust statistical analyses are the major strength of the study.

The QUALICOPC framework was designed to study and compare primary health‐care properties and patient perceptions between countries, not patient enablement in itself. Therefore, the measurement was a single‐item question and not the ‘gold standard’ Patient Enablement Instrument with six questions. Nonetheless, this question seems to be adequate for identifying patients with low enablement scores.^16^ Furthermore, not all potential factors could be included in the analyses. For example, more detailed data of GP personal characteristics or actual time consumed in the consultation were not available. In addition, despite the large amount of data, loss of observations due to missing values—a common challenge with a logistic regression analysis—and merging several data sets collected in separate studies caused some loss of data. Additionally, there could be a circularity phenomenon for all perceptual patient variables, for example patient satisfaction and trust in doctors. Lastly, since this is a study about associations, conclusions in terms of causality cannot be drawn.

## 7 CONCLUSIONS

In the international context, cultural dimensions and GP and practice characteristics explain patient enablement variation between countries to a rather large extent. Patient and—to some extent—practice characteristics seem to explain a minor part of practice variation. In contrast, structural elements of health care show no significant associations. In addition, several independent variables seem to be associated with patient enablement. GPs and researchers should be aware of the potential importance of cultural aspects, particularly when comparing health survey results between countries and adopting measurements across countries.

## 8 CLINICAL IMPLICATIONS

Enablement is a goal worth pursuing for all patients, in order to ensure an experience of coping and understanding. Doctors should aim to strengthen patient enablement, not only as a measure of quality but also as an important issue in itself. Recognizing factors that associate with lower enablement—for example patients’ lower self‐perceived health—may help doctors to focus on the patients who may need more attention or actions in order to achieve enablement. Practising skills related to patient‐centred consultation and patient involvement, as well as improving continuity and access to care, may contribute to better patient enablement across countries.

## ETHICAL APPROVAL AND CONSENT TO PARTICIPATE

Ethical approval was acquired in accordance with the legal requirements in each country. Both GP and patient surveys were carried out anonymously. Although a standardized data collection procedure across all countries was strongly recommended and strived for, in the actual data collection strategy cultural and ethical requirements for each country were taken into account.

## Supporting information

File S1‐S3

## Data Availability

The data are available upon reasonable request, via the corresponding author.
