# Supplementary material for: Patient enablement after a consultation with a general practitioner—Explaining variation between countries, practices and patients
Source: Health Expect. 2020 Jun 29;23(5):1129–43. doi: 10.1111/hex.13091 (PMC7696125; doi:10.1111/hex.13091)
Supplement: Supplementary file 1 — File S1‐S3 [file HEX-23-1129-s001.docx]

**Additional file 1. Operationalization of variables**

|  |  |  | Operationalization | |
| --- | --- | --- | --- | --- |
| **PATIENT-LEVEL VARIABLES** | | |  |  |
| **Patient characteristics** | | |  |  |
|  | Age |  | What is your year of birth? | |
|  | Gender |  | Are you male or female? | |
|  | Household income | | Compared to the average household income of this country, would you say your household’s income is: below average/average/above average? | |
|  | Education |  | What is the highest level of education that you achieved? No qualifications obtained, pre-primary education, primary education or lower secondary level education/upper secondary level of education/post-secondary, non-tertiary education or higher | |
|  | Ethnicity |  | Where were you born? And Where was your mother born? In this country/In another EU country/In a European country outside the EU/North America, Australia or New Zealand/In another country | |
|  | Language skills | | How well do you speak an official language of this country? Fluently/native speaker level vs. Sufficiently/Moderately/Poorly/Not at all | |
|  | Chronic disease | | Do you have a longstanding disease or condition such as high blood pressure, diabetes, depression, asthma? Yes/No | |
|  | Self-perceived health | | How would you describe your own health in general? Very good/Good vs. Fair/Poor | |
|  | Reason for consultation | |  | |
|  |  | | Because I was ill or did not feel well | |
|  |  | | For a medical check-up | |
|  |  | | To get a prescription/referral/medical certificate | |
|  |  | | For a second opinion/other reason | |
| **Patient-perceived consultation variables** | | | |  |
|  | Involvement |  | The doctor involved me in making decisions about treatment, no/yes | |
|  | Trust |  | In general, doctors can be trusted, strongly disagree/disagree vs. agree/strongly agree | |
|  | Patient satisfaction (scale variable) | |  | |
|  |  | | The doctor took sufficient time, no/yes | |
|  |  |  | I would recommend this doctor to a friend or relative, no/yes | |
|  |  |  | The doctor asked about possible other problems besides the one I came in for, no/yes | |
|  |  |  | This doctor doesn’t just deal with medical problems but can also help with personal problems, no/yes | |
|  |  |  | The doctor was polite, no/yes | |
|  |  |  | The doctor asked questions about my health problem, no/yes | |
|  |  |  | People were polite and helpful at the reception desk, no/yes/don’t know† | |
|  | Continuity of care (scale variable) | | |  |
|  |  |  | Do you have your own doctor? Yes, the one I just visited/Yes, but another doctor in this practice or centre OR Yes, but another doctor somewhere else/No, I do not have my own doctor | |
|  |  |  | This doctor knows my medical background, no/yes/don’t know† | |
|  |  |  | This doctor knows my living situation | |
|  | Perception of access to care (scale variable) | | | |
|  |  |  | The opening hours are too restricted, no/yes/don’t know† | |
|  |  |  | If I need a home visit I can get one, no/yes/don’t know† | |
|  |  |  | The practice is too far away from where I live or work, no/yes/don’t know† | |
|  |  |  | When I called this practice, I had to wait too long to speak to someone, no/yes/don’t know† | |
|  |  |  | I know how to get evening, night and weekend services, no/yes/don’t know) † | |
|  | Previous negative experience: discrimination (scale variable) | | | |
|  |  |  | The doctor or staff acted negatively to you (In the past 12 months), no/yes | |
|  |  |  | Other patients were treated better than you (In the past 12 months), no/yes | |
|  |  |  | The doctor or staff showed disrespect because of your ethnic background (In the past 12 months), no/yes | |
|  |  |  | The doctor or staff showed disrespect because of your gender (In the past 12 months), no/yes | |
|  | Propensity to seek care from GP (scale variable) | | |  |
|  |  |  | Severe complaints: How important would it be for you to see the doctor if you had…. (7 alternatives from weight loss to severe worries) | |
|  |  |  | Minor complaints: Do you expect to benefit from a visit to a GP if you had… (9 alternatives from stomach problems to feeling nauseous) | |
|  | Communication (scale variable) | |  | |
|  |  |  | The doctor was polite, no/yes | |
|  |  |  | The doctor listened carefully to me, no/yes | |
|  |  |  | The doctor hardly looked at me when we talked, no/yes (reverse coded) | |
|  |  |  | The doctor asked questions about my health problem, no/yes | |
|  |  |  | I couldn’t really understand what the doctor was trying to explain, no/yes (reverse coded) | |
| **PRACTICE-LEVEL VARIABLES** | | |  |  |
| GP characteristics | |  |  |  |
|  | GP’s age |  | What is your year of birth? | |
|  | GP’s gender |  | Are you male or female? | |
| Practice characteristics | | |  |  |
|  | Practice location | | How would you characterize the place you are currently practising? Large (inner) city vs. suburbs/(Small) town vs. Mixed urban-rural/Rural | |
|  | GP’s accommodation | | Do you work alone or in shared accommodation? Alone vs. with other GPs/with medical specialists in shared accommodation | |
|  | GP’s remuneration | | As a GP, are you self-employed or in salaried employment? Salaried employment with centre or authority/Salaried employment with other GP vs. Self-employed with contract(s) with health service, insurance or authority/Self-employed without contract | |
| Consultation length | | |  |  |
|  | Mean time for consultation | | How long does a regular patient consultation in your office usually take? (minutes) | |
|  | Mean number of face-to-face consultation per day | | How many patient contacts do you have on a normal working day? (face-to-face in your office, number) | |
| Work-related stress | | |  |  |
|  | Perceived work-related stress | | To what extent do you agree with the following statements: I have too much stress in my current job; Strongly agree/agree vs. Disagree/strongly disagree | |
|  | Effort-reward balance | | To what extent do you agree with the following statements: In my work, there is a good balance between effort and reward; Strongly agree/agree vs. Disagree/strongly disagree | |
| Collaboration with other providers | | | How often do you meet face-to-face with the following professionals (either professionally or socially): Other GP/Practice nurse/Ambulatory medical specialist/Hospital medical specialist/Pharmacist/Home care nurse/Midwife/Physiotherapist/Social worker/Dietician? Seldom or never/Every 1–3 months/More than once a month | |
| Skills mix in workplace | | | Which of the following disciplines are covered in your practice/centre? Receptionist or medical secretary/Practice nurse/Community or home care nurse/Psychiatric nurse/Nurse practitioner (function between physician and nurse/Assistant for laboratory work/Manager of the centre or practice (not a physician)/Midwife/Physiotherapist/Dentist/Pharmacist/Social worker | |
| Possibilities to perform minor technical procedures | | | To what extent are the following activities carried out in your practice population by you (or your staff) and not by a medical specialist? Wedge resection of ingrown toenail/Removal of sebaceous cyst from hairy scalp/Wound suturing/Excision of warts/Insertion of IUD/fundoscopy/Joint injection/Strapping an ankle/Cryotherapy (warts)/Setting up an intravenous infusion? (Almost) always/Usually/Occasionally/Seldom or never | |
| **COUNTRY-LEVEL VARIABLES** | | |  |  |
| Gatekeeping | |  | See *Kringos DS, Boerma WGW, Hutchinson A, Saltman RB, editors. Building primary care in a changing Europe: Case studies. European Observatory on Health Systems and Policies; (c) World Health Organization 2015; 2015.* | |
| PHC structure—PHAMEU variables | | | See *Kringos DS, Boerma WGW, Hutchinson A, Saltman RB, editors. Building primary care in a changing Europe: Case studies. European Observatory on Health Systems and Policies; (c) World Health Organization 2015; 2015.* | |
|  | Governance |  |  |  |
|  | Economic condition | |  |  |
|  | Workforce development | |  |  |
|  | Total structure | |  |  |
| Cultural Dimensions—VSM variables | | | See *Hofstede G, Jan Hofstede G, Minkov M. Cultures and Organizations: Software of the Mind, Intercultural Cooperation and Its Importance for Survival. Cultures and Organizations. 2010.* | |
|  | Power Distance | |  |  |
|  | Individualism vs. Collectivism | |  |  |
|  | Masculinity vs. Femininity | |  |  |
|  | Uncertainty Avoidance | |  |  |
|  | Long-Term vs. Short-Term Orientation | | |  |
|  | Indulgence vs. Restraint | |  |  |
| QUALICOPC Patient Values | | | See *Schäfer WLA, Boerma WGW, Kringos DS, De Ryck E, Greß S, Heinemann S, et al. Measures of quality, costs and equity in primary health care instruments developed to analyse and compare primary care in 35 countries. Qual Prim Care. 2013;21(2):67–79.* | |
|  | | | How important are the following to you: Not important/Somewhat important/Important/Very important | |
|  | Enablement |  | That I feel able to cope better with my health problem/illness after this visit | |
|  | Treating as a person | | That the doctor treats me as a person and not just a medical problem | |
|  | Knowing the background | | That this doctor knows important information about my medical background | |

† don’t knows combined with no responses

**Additional file 2. Hofstede’s Cultural Dimensions.**

**Power Distance (PDI)**

- ‘as the extent of which the less powerful members of institutions and organizations within a country expect and accept that power is distributed unequally’. ^48^
- In general, in countries with large Power Distance, for example inequalities among people is expected and desired; parents teach children obedience instead of treating them as equals; strictly hierarchical structures are more common. ^48^
- In terms of health care, in countries with large PDI, consultations are shorter and more controlled by doctors; doctors and patients have fixed roles. ^57^

**Individualism versus Collectivism (IDV)**

- ‘Individualism pertains to societies in which the ties between individuals are loose: everyone is expected to look after him- or herself and his or her immediate family. Collectivism [..] pertains to societies in which people from birth onward are integrated into strong, cohesive in-groups, which throughout people’s lifetimes continue to protect them in exchange for unquestioning loyalty’.^48^
- In general, in countries with strong Individualism, people think ‘I’ instead of ‘we’; independence of others and self-supporting lifestyles are encouraged, laws and rights are supposed to be the same for all; autonomy is the ideal (over patriotism), and so on. ^48^
- The more individualistic a country, the more affective behaviour is shown by physicians, and the more flexible the roles of doctors and patients. ^57^

**Masculinity versus Femininity (MAS)**

- ‘A society is called masculine when emotional gender roles are clearly distinct; men are supposed to be assertive, tough, and focused on material success, whereas women are supposed to be modest, tender and concerned with the quality of life. A society is called feminine when emotional gender roles overlap: both men and women are supposed to be modest, tender and concerned with the quality of life’. ^48^
- In general, in countries with strong Femininity, there are the same standards for both sexes; men and women study the same subjects; people work in order to live (not vice versa); welfare society idea (help for the needy instead of support for the strong). ^48^

**Uncertainty Avoidance (UAI)**

- ‘as an extent to which the members of a culture feel threatened by ambiguous and unknown situations’. ^48^
- In general, in countries with weak Uncertainty Avoidance, Uncertainty is a normal feature of life; low stress and anxiety; fewer people feel unhappy; teachers may say ‘I don’t know’; time is a framework for orientation; few and general laws.
- In countries with strong uncertainty avoidance, physicians were less satisfied with their jobs; had less eye contact with patients and were less open to patients ^57^ and prescribed more antibiotics. ^58^

**Long-Term versus Short-Term Orientation (LTOWVS)**

- ‘Long-term orientation stands for the fostering of virtues toward future rewards—in particular, perseverance and thrift. Its opposite pole, short-term orientation, stands for the fostering of virtues related to the past and present—in particular, respect for tradition, preservation of “face” and fulfilling social obligations’. ^48^
- In general, in countries with long-term orientation, there is respect of circumstances instead of traditions; priority is given to common sense; appeal of pragmatism instead of fundamentalism. ^48^

**Indulgence versus Restraint (IVR)**

- ‘Indulgence stands for a tendency to allow relatively free gratification of basic and natural human desires enjoying life and having fun. Its opposite pole, restraint, reflects a conviction that such a gratification needs to be curbed and regulated by strict social norms’. ^48^
- The Indulgence dimension is associated with higher optimism and better subjective health. ^48^
- In general, in countries with high Indulgence, there is higher importance placed on leisure and having friends; a looser society; more satisfying family life; higher percentage of obese people (in wealthy countries); loosely prescribed gender roles.
- In countries with higher Indulgence, patients appreciate more doctor-patient roles and responsibilities. ^59^

**Additional file 3.** Results of multilevel logistic regression analyses (Models 1–3): the odds ratio (OR) to respond negatively to dependent question ‘After this visit, I feel I can cope better with my health problem than before the appointment’. The variation distribution across country and practice; the proportions of explained variances.

|  |  |  |  |  |  |  |  |  |  |  |  |  |  |  |
| --- | --- | --- | --- | --- | --- | --- | --- | --- | --- | --- | --- | --- | --- | --- |
|  |  |  | | Model 1 | | |  | Model 2 | | | | Model 3 - Final model | | |
|  |  |  |  | N = 53,738 | | |  | N = 48,416 | | | | N = 48,416 | | |
|  |  |  |  | OR | p | 95%CI |  | OR | p | 95%CI |  | OR | p | 95%CI |
| **PATIENT-LEVEL VARIABLES** | | |  |  |  |  |  |  |  |  |  |  |  |  |
| **Patient characteristics** | | |  |  |  |  |  |  |  |  |  |  |  |  |
| Patient’s age | | |  |  |  |  |  |  |  |  |  |  |  |  |
|  | under 40 years | |  | ref |  |  |  |  |  |  |  |  |  |  |
|  | 40-64 years | |  | 0.84 | <0.001 | 0.79-0.89 |  | 0.84 | <0.001 | 0.7-0.89 |  | 0.84 | <0.001 | 0.79-0.89 |
|  | over 65 years | |  | 0.80 | <0.001 | 0.71-0.88 |  | 0.81 | <0.001 | 0.73-0.90 |  | 0.81 | <0.001 | 0.73-0.90 |
| Patient’s gender | | |  |  |  |  |  |  |  |  |  |  |  |  |
|  | Male |  |  | ref |  |  |  |  |  |  |  |  |  |  |
|  | Female |  |  | 0.88 | <0.001 | 0.84-0.93 |  | 0.87 | <0.001 | 0.83-0.92 |  | 0.87 | <0.001 | 0.83-0.92 |
| Ethnicity | |  |  |  |  |  |  |  |  |  |  |  |  |  |
|  | Native |  |  | ref |  |  |  |  |  |  |  |  |  |  |
|  | Second-generation immigrant | | | 1.06 | 0.30 | 0.94-1.19 |  | 1.07 | 0.28 | 0.95-1.21 |  | 1.07 | 0.28 | 0.95-1.21 |
|  | First-generation immigrant | | | 0.88 | 0.02 | 0.79-0.98 |  | 0.91 | 0.07 | 0.82-1.01 |  | 0.90 | 0.07 | 0.81-1.01 |
| Language skills | | |  |  |  |  |  |  |  |  |  |  |  |  |
|  | Fluently/native speaker level | | | ref |  |  |  |  |  |  |  |  |  |  |
|  | Sufficiently/Moderately/Poorly/Not at all | | | 1.00 | 0.95 | 0.80-1.08 |  | 1.00 | 0.96 | 0.93-1.08 |  | 1.01 | 0.89 | 0.93-1.09 |
| Chronic disease | | |  |  |  |  |  |  |  |  |  |  |  |  |
|  | No |  |  | ref |  |  |  |  |  |  |  |  |  |  |
|  | Yes |  |  | 0.98 | 0.58 | 0.93-1.08 |  | 0.98 | 0.61 | 0.93-1.05 |  | 0.98 | 0.61 | 0.93-1.05 |
| Self-perceived health | | |  |  |  |  |  |  |  |  |  |  |  |  |
|  | Very good/good | |  | ref |  |  |  |  |  |  |  |  |  |  |
|  | Fair/poor |  |  | 1.28 | <0.001 | 1.21-1.36 |  | 1.29 | <0.001 | 1.22-1.37 |  | 1.29 | <0.001 | 1.22-1.37 |
| Education | |  |  |  |  |  |  |  |  |  |  |  |  |  |
|  | No/primary level | |  | ref |  |  |  |  |  |  |  |  |  |  |
|  | Upper secondary level | |  | 1.04 | 0.25 | 0.97-1.11 |  | 1.04 | 0.24 | 0.97-1.12 |  | 1.04 | 0.25 | 0.97-1.11 |
|  | Post-secondary level | |  | 1.09 | 0.01 | 1.01-1.18 |  | 1.09 | 0.03 | 1.01-1.18 |  | 1.09 | 0.03 | 1.01-1.18 |
| Household income | | |  |  |  |  |  |  |  |  |  |  |  |  |
|  | Below average | |  | ref |  |  |  |  |  |  |  |  |  |  |
|  | Around average | |  | 0.90 | <0.001 | 0.85-0.95 |  | 0.91 | 0.004 | 0.86-0.97 |  | 0.91 | 0.003 | 0.86-0.97 |
|  | Above average | |  | 0.91 | 0.04 | 0.83-0.99 |  | 0.93 | 0.15 | 0.85-1.02 |  | 0.93 | 0.15 | 0.85-1.02 |
| Occupation | | |  |  |  |  |  |  |  |  |  |  |  |  |
|  | Working, including civil service and self-employment | | | ref |  |  |  |  |  |  |  |  |  |  |
|  | Retired |  |  | 0.93 | 0.14 | 0.85-1.02 |  | 0.93 | 0.13 | 0.85-1.02 |  | 0.93 | 0.13 | 0.85-1.02 |
|  | Student, unemployed, unable to work, mainly homemaker | | | 1.05 | 0.10 | 0.99-1.12 |  | 1.07 | 0.04 | 1.00-1.14 |  | 1.07 | 0.04 | 1.00-1.14 |
| Consultation reason | |  |  |  |  |  |  |  |  |  |  |  |  |  |
|  | Illness |  |  | ref |  |  |  |  |  |  |  |  |  |  |
|  | Medical check-up |  |  | 1.04 | 0.28 | 0.97-1.10 |  | 1.06 | 0.08 | 0.99-1.14 |  | 1.06 | 0.08 | 0.99-1.13 |
|  | Prescription, referral or certificate | |  | 1.38 | <0.001 | 1.30-1.48 |  | 1.40 | <0.001 | 1.31-1.51 |  | 1.40 | <0.001 | 1.31-1.51 |
|  | Other |  |  | 1.18 | <0.001 | 1.10-1.26 |  | 1.12 | <0.001 | 1.11-1.29 |  | 1.20 | <0.001 | 1.11-1.29 |
|  | | |  |  |  |  |  |  |  |  |  |  |  |  |
| **Patient perceptions of consultation** | | |  |  |  |  |  |  |  |  |  |  |  |  |
| Patient involvement | | |  |  |  |  |  |  |  |  |  |  |  |  |
|  | No | | | ref |  |  |  |  |  |  |  |  |  |  |
|  | Yes |  |  | 0.58 | <0.001 | 0.55-0.62 |  | 0.58 | <0.001 | 0.54-0.62 |  | 0.58 | <0.001 | 0.54-0.62 |
| Trust in doctors | | |  |  |  |  |  |  |  |  |  |  |  |  |
|  | Agree |  |  | ref |  |  |  |  |  |  |  |  |  |  |
|  | Disagree |  |  | 1.57 | <0.001 | 1.41-1.75 |  | 1.58 | <0.001 | 1.41-1.78 |  | 1.58 | <0.001 | 1.41-1.77 |
| Patient satisfaction (scale with 7 variables)† | | | | 0.53 | <0.001 | 0.52-0.55 |  | 0.54 | <0.001 | 0.51-0.55 |  | 0.54 | <0.001 | 0.52-0.56 |
| Continuity of care (scale with 3 variables) | | | | 0.70 | <0.001 | 0.68-0.73 |  | 0.70 | <0.001 | 0.67-0.72 |  | 0.70 | <0.001 | 0.67-0.73 |
| Perceptions of access to care (scale with 5 variables) | | | | 0.85 | <0.001 | 0.82-0.88 |  | 0.84 | <0.001 | 0.80-0.87 |  | 0.84 | <0.001 | 0.81-0.87 |
| No previous discrimination (scale with 4 variables) | | | | 0.97 | 0.01 | 0.94-0.99 |  | 0.96 | 0.002 | 0.93-0.98 |  | 0.96 | 0.002 | 0.93-0.98 |
| Propensity to seek care (severe complains, scale) | | | | 0.86 | <0.001 | 0.84-0.88 |  | 0.86 | <0.001 | 0.83-0.88 |  | 0.86 | <0.001 | 0.83-0.88 |
| Propensity to seek care (minor complains, scale) | | | | 0.89 | <0.001 | 0.87-0.92 |  | 0.89 | <0.001 | 0.87-0.91 |  | 0.89 | <0.001 | 0.86-0.91 |
| Perception of communication (scale with 5 variables) | | | | 1.05 | 0.01 | 1.01-1.08 |  | 1.03 | 0.08 | 0.97-1.07 |  | 1.03 | 0.07 | 0.99-1.07 |
|  | |  |  |  |  |  |  |  |  |  |  |  |  |  |
| **GP-LEVEL variables** | |  |  |  |  |  |  |  |  |  |  |  |  |  |
| GP’s age | |  |  |  |  |  |  |  |  |  |  |  |  |  |
|  | 21-39 |  |  |  |  |  |  | ref |  |  |  |  |  |  |
|  | 40-64 |  |  |  |  |  |  | 1.05 | 0.27 | 0.96-1.15 |  | 1.05 | 0.29 | 0.96-1.15 |
|  | 65 and over | |  |  |  |  |  | 1.09 | 0.29 | 0.93-1.29 |  | 1.09 | 0.32 | 0.92-1.28 |
| GP’s gender | |  |  |  |  |  |  |  |  |  |  |  |  |  |
|  | male |  |  |  |  |  |  | ref |  |  |  |  |  |  |
|  | female |  |  |  |  |  |  | 0.98 | 0.51 | 0.91-1.05 |  | 0.98 | 0.53 | 0.92-1.05 |
| GP’s practice location | | |  |  |  |  |  |  |  |  |  |  |  |  |
|  | Big inner city | |  |  |  |  |  | ref |  |  |  |  |  |  |
|  | Suburbs or small town | |  |  |  |  |  | 1.07 | 0.08 | 0.99-1.17 |  | 1.08 | 0.07 | 0.99-1.17 |
|  | Urban-rural or rural | |  |  |  |  |  | 1.12 | 0.01 | 1.03-1.22 |  | 1.12 | 0.01 | 1.03-1.22 |
| GP’s accommodation | | |  |  |  |  |  |  |  |  |  |  |  |  |
|  | Solo practice | |  |  |  |  |  | ref |  |  |  |  |  |  |
|  | Duo or group practice | |  |  |  |  |  | 0.97 | 0.50 | 0.89-1.05 |  | 0.98 | 0.58 | 0.91-1.06 |
| GP’s remuneration | | |  |  |  |  |  |  |  |  |  |  |  |  |
|  | Salaried |  |  |  |  |  |  | ref |  |  |  |  |  |  |
|  | Self-employed | |  |  |  |  |  | 1.13 | 0.04 | 1.01-1.26 |  | 1.11 | 0.08 | 0.99-1.24 |
|  | Mixed |  |  |  |  |  |  | 0.94 | 0.71 | 0.66-1.33 |  | 0.92 | 0.63 | 0.64-1.30 |
| Mean consultation time (GP declared) | | |  |  |  |  |  |  |  |  |  |  |  |  |
|  | 0-4 minutes | |  |  |  |  |  | ref |  |  |  |  |  |  |
|  | 5-9 minutes | |  |  |  |  |  | 0.82 | 0.21 | 0.60-1.12 |  | 0.82 | 0.21 | 0.60-1.11 |
|  | 10-14 minutes | |  |  |  |  |  | 0.82 | 0.19 | 0.60-1.11 |  | 0.82 | 0.19 | 0.60-1.11 |
|  | 15-29 minutes | |  |  |  |  |  | 0.76 | 0.08 | 0.56-1.04 |  | 0.76 | 0.09 | 0.56-1.04 |
|  | over 30 minutes | |  |  |  |  |  | 0.71 | 0.05 | 0.50-1.00 |  | 0.71 | 0.05 | 0.50-1.01 |
| Mean number of face-to face consultations (GP declared) | | |  |  |  |  |  |  |  |  |  |  |  |  |
|  | 0-14 |  |  |  |  |  |  | ref |  |  |  |  |  |  |
|  | 15-29 |  |  |  |  |  |  | 0.91 | 0.17 | 0.80-1.04 |  | 0.91 | 0.19 | 0.80-1.04 |
|  | 30-44 |  |  |  |  |  |  | 0.90 | 0.17 | 0.78-1.04 |  | 0.91 | 0.18 | 0.78-1.05 |
|  | 45 or more | |  |  |  |  |  | 0.82 | 0.02 | 0.70-0.97 |  | 0.82 | 0.02 | 0.70-0.97 |
| GP-perceived work-related stress | | |  |  |  |  |  |  |  |  |  |  |  |  |
|  | Agree |  |  |  |  |  |  | ref |  |  |  |  |  |  |
|  | Disagree |  |  |  |  |  |  | 1.02 | 0.45 | 0.96-1.10 |  | 1.03 | 0.43 | 0.96-1.10 |
| GP-perceived effort-reward imbalance | | | |  |  |  |  |  |  |  |  |  |  |  |
|  | Agree |  |  |  |  |  |  | ref |  |  |  |  |  |  |
|  | Disagree |  |  |  |  |  |  | 1.00 | 0.99 | 0.94-1.07 |  | 1.00 | 1.00 | 0.93-1.07 |
|  | Collaboration with other providers (scale) |  |  |  |  |  |  | 1.02 | 0.42 | 0.98-1.06 |  | 1.02 | 0.38 | 0.98-1.06 |
|  | Skill mix (scale) |  |  |  |  |  |  | 0.95 | 0.26 | 0.88-1.03 |  | 0.96 | 0.25 | 0.88-1.03 |
|  | Technical procedures (scale) | |  |  |  |  |  | 1.00 | 0.88 | 0.94-1.05 |  | 1.00 | 0.98 | 0.95-10.6 |
|  | | |  |  |  |  |  |  |  |  |  |  |  |  |
| **COUNTRY-LEVEL variables** | | |  |  |  |  |  |  |  |  |  |  |  |  |
|  | Individualism vs. Collectivism (towards individualism) | | |  |  |  |  |  |  |  |  | 1.11 | 0.26 | 0.93-1.32 |
|  | Uncertainty Avoidance (towards uncertainty avoiding) | | |  |  |  |  |  |  |  |  | 0.88 | 0.15 | 0.74-1.04 |
|  | Long-term Orientation (towards short-term orientation) | | |  |  |  |  |  |  |  |  | 1.27 | <0.001 | 1.11-1.46 |
|  |  |  |  |  |  |  |  |  |  |  |  |  |  |  |
|  | |  | |  |  |  |  |  |  |  |  |  |  |  |
| **MODEL VARIANCES** | | Null model | |  | Model 1 |  |  |  | Model 2 |  |  | Model 3—Final Model | | |
|  | Country variance | 0.2598 | |  | 0.2573 |  |  |  | 0.2230 |  |  |  | 0.1284 |  |
|  | Practice variance | 0.661 | |  | 0.5264 |  |  |  | 0.5398 |  |  |  | 0.5398 |  |
|  |  |  | |  |  |  |  |  |  |  |  |  |  |  |
|  | Country variance explained, % |  | |  | 0.96 |  |  |  | 14.2 |  |  |  | 50.6 |  |
|  | Practice variance explained, % |  | |  | 20.3 |  |  |  | 18.4 |  |  |  | 18.4 |  |
|  |  |  | |  |  |  |  |  |  |  |  |  |  |  |
|  | MOR (median odds ratio) for country level | 1.63 | |  | 1.62 |  |  |  | 1.56 |  |  |  | 1.41 |  |
|  | MOR for practice level | 2.17 | |  | 2.00 |  |  |  | 2.01 |  |  |  | 2.01 |  |
|  |  |  | |  |  |  |  |  |  |  |  |  |  |  |

Note: Statistically significant ORs that diminish or increase the probability of responding negatively to the dependent question are marked with green and yellow, respectively.

†: scale variables are presented as z-scores
